# Isolated Growth Hormone Deficiency Type 2 due to a novel *GH1* Mutation: A Case Report

**DOI:** 10.4274/jcrpe.galenos.2019.2018.0305

**Published:** 2019-11-22

**Authors:** Ahmad Kautsar, Jan M. Wit, Aman Pulungan

**Affiliations:** 1 University of Indonesia, Cipto Mangunkusumo Hospital, Department of Child Health, Jakarta, Indonesia; 2 Leiden University Medical Center, Department of Paediatrics, Leiden, The Netherlands

**Keywords:** Growth hormone, GH1, short stature, isolated growth hormone deficiency

## Abstract

Isolated growth hormone (GH) deficiency (IGHD) type 2 is a rare autosomal dominant disorder characterized by severe short stature with low GH level. Timely diagnosis is important for optimal results of recombinant human GH (rhGH) treatment and detection of additional pituitary deficiencies in affected relatives. A male child presented at the age of one year with severe, proportionate short stature [-4.9 standard deviation score (SDS)] and with a normal body mass index (-1.1 SDS). Physical examination revealed frontal bossing, midfacial hypoplasia, normal external genitalia and no dysmorphic features. Paternal and maternal heights were -6.1 and -1.9 SDS. Serum insulin-like growth factor-1 (IGF-1) and IGF-binding protein-3 were undetectable and the peak GH concentration by clonidine stimulation test was extremely low (0.18 ng/mL). Brain magnetic resonance imaging showed anterior pituitary hypoplasia. Genetic analysis identified a novel heterozygous mutation (c.291+2T>G) expected to lead to splicing out exon 3 of GH1. rhGH from age 2.4 years led to appropriate catch-up. In conclusion, we identified a novel *GH1* gene mutation in an infant with classical IGHD type 2 presentation.

**What is already known on this topic?**

Dominantly inherited isolated growth hormone deficiency (IGHD) can be caused by multiple defects of the *GH1* gene. Affected individuals show a good growth response to recombinant human GH and can develop multiple pituitary deficiency.

**What this study adds?**

A novel *GH1* gene mutation was found in an Indonesian infant with the classical presentation of IGHD type 2.

## Introduction

Growth hormone (GH) deficiency (GHD) is characterized by decreased GH secretion as assessed by one or two GH provocation tests in addition to low serum insulin-like growth factor-1 (IGF-I) and IGF-binding protein-3 (IGFBP-3) concentrations and clinical features including linear growth failure, typical features at physical examination and bone age retardation (1). GHD can be either isolated GHD (IGHD) or part of multiple pituitary hormone deficiency (MPHD) and can be congenital or acquired. The reported incidence of congenital GHD is 1 in 4,000 to 1 in 10,000 live births with male predominance (2,3).

When IGHD is suspected, further evaluation is urgently needed (4). Establishing the diagnosis is a multistep process involving a careful medical history, detailed physical examination including accurate measurements of stature and analysis of the growth curve, biochemical testing, pituitary imaging and genetic screening in severe and/or familial cases (4,5,6,7,8,9).

Genetic causes of IGHD can be found in 3-30% of patients and are typically classified into four types according to the inheritance pattern: autosomal recessive inheritance (IGHD types 1A and 1B), autosomal dominant (IGHD type 2), and X-linked inheritance (IGHD type 3) (2,3,5). Mutations of the genes encoding GH (*GH1*), GHRH receptor (*GHRHR*), the GH secretagogue receptor (*GHSR*) and several transcription factors involved in pituitary development have been described to cause IGHD (5,10).

Here we report a case of genetically proven, autosomal dominant IGHD type 2 caused by a novel mutation of *GH1* at a position where previously two other mutations have been found (10).

## Case Report

A male infant, the 0.99 year old son of non-consanguineous parents was referred to our pediatric endocrinology clinic because of severe short stature. His father’s height was 132 cm [-6.1 standard deviation score (SDS)] and maternal height was 151 cm (-1.86 SDS). Pregnancy and the perinatal period were uneventful. Birth weight and length were 3.3 kg and 48 cm after 38 weeks of pregnancy (-0.1 and -1.0 SDS, respectively). There were no indications of any chronic disease and psychomotor development was normal.

**Figure 1:**
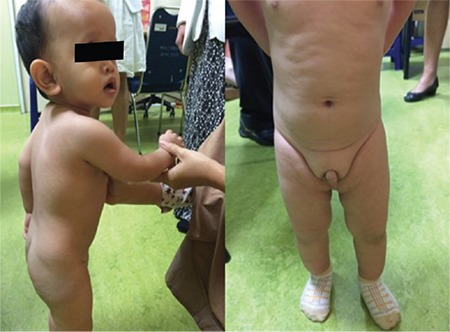
Characteristic clinical features of the patient. Frontal bossing, midfacial hypoplasia, lobulated subcutaneous fat and normal genitalia are noted

Length and weight with SDS calculations based on the World Health Organization growth charts at first presentation were 64 cm (-4.9 SDS) and 6.3 kg (-4.8 SDS), respectively (11), body mass index was 15.4 kg/m^2^ (-1.1 SDS) and head circumference 44 cm (-1.6 SDS). Physical examination revealed frontal bossing, midfacial hypoplasia, normal external genitalia and no dysmorphic features (Figure 1). Further anthropometric data revealed a proportionate short stature with a sitting height/height ratio of 0.65 (0.1 SDS) (12). The growth velocity foregoing the first observation was 3 cm over six months (-3.5 SDS) (11). Bone age was 6 months at a chronological age of 1.0 year.

Laboratory examination revealed a normal free thyroxine (fT4) level (fT4, 1.23 ng/dL) and thyroid stimulating hormone (TSH) (TSH; 2.74 µU/mL) and undetectable levels of IGF-1 (<25 ng/mL) and IGFBP-3 (<0.5 mg/L). The patient’s father also had a low serum IGF-I (<25 ng/mL).

**Figure 2:**
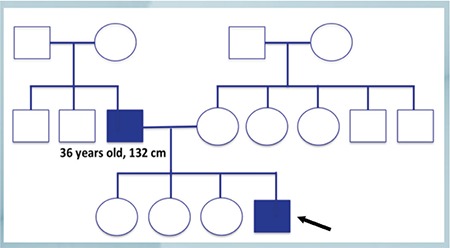
The pedigree of the family of the index patient with autosomal dominant type 2 growth hormone deficiency. Filled squares indicate affected members [the index patient (arrow) and the father]

The pedigree of the family is shown in Figure 2. The heights of the paternal grandfather and grandmother were reported as approximately 165 cm (≈-1.6 SDS) and 150 cm (-2.0 SDS), respectively.

**Figure 3:**
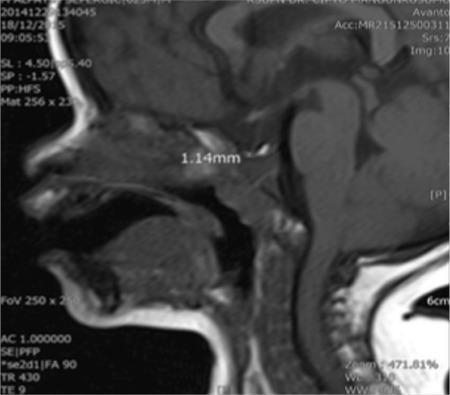
Brain magnetic resonance of the index case, demonstrating anterior pituitary hypoplasia

**Figure 4:**
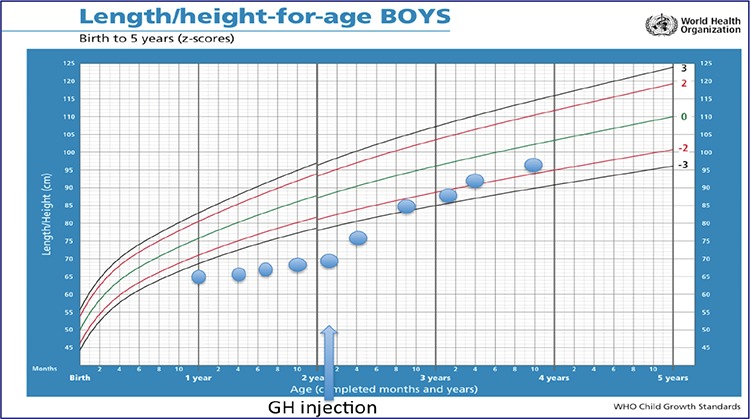
Height data of the patient plotted on the World Health Organization growth chart. The arrow indicates the beginning of recombinant human growth hormone injections GH: growth hormone

The patient then underwent a GH stimulation test using clonidine 0.15 mg/m^2^. Peak GH level was extremely low (0.18 ng/mL). An magnetic resonance imaging (MRI) of the brain showed anterior pituitary hypoplasia (Figure 3). Due to financial constraints it took more than a year before recombinant human GH (rhGH) (Saizen, Merck-Serono) replacement therapy could be started at the age of 2 years and 5 months at a daily dose of 20-24 mg/kg body weight. This resulted in a appropriate catch-up growth (Table 1, Figure 4). Growth velocity after 1.5 years of treatment was 9.5 cm/year over a 13 month period. Screening for deficiencies of other pituitary hormones including follicle stimulating hormone (FSH), luteinizing hormone (LH), TSH and morning cortisol, showed normal results. Screening the father for other pituitary and related hormones including FSH, LH, testosterone, fT4, TSH, prolactin, adrenocorticotropin hormone (ACTH) and cortisol, also yielded normal results.

Sanger sequencing of *GH1* was performed in the laboratory of Centogene AG (Rostock, Germany) and showed a novel heterozygous mutation (c.291+2T>G) expected to lead to splicing out of exon 3. Mutation analysis of the father’s DNA has not been performed, but the extremely short stature and low IGF-1 make it highly likely that he carries the same mutation, which appears to be *de novo* according to the normal heights of the paternal grandparents and the father’s brothers.

All clinical investigations were conducted in accordance with the guidelines by the Declaration of Helsinki. The parents gave informed consent to clinical and genetic studies, as well as for publication of the clinical information and pictures.

## Discussion

In this report we describe a novel splice site mutation of *GH1* leading to severe short stature in the index patient and his father, a characteristic finding for type 2 IGHD. No other relatives with severe short stature are known in this family, so we have assumed that the mutation occurred *de novo* in the patient’s father. The mutation is located at a base known to be vital for correct splicing, since previous mutations c.291+2T>A and >C have been discovered with an autosomally inherited and similarly severe phenotype (13,14,15), with lower GH peaks upon provocation compared with those with missense mutations (13). The hypoplastic anterior pituitary in the patient is consistent with previous observations reported in 60% of patients with splice spite mutations (13). The severe IGHD with early onset is thought to be caused by a disturbance of GH storage and secretion due to misfolded, mutant GH (16).

The combination of early-onset severe proportionate growth failure, bone age delay and classical physical signs (midfacial hypoplasia and frontal bossing) makes the *a priori* likelihood of congenital GHD very high. This should always lead to laboratory testing (serum IGF-I and IGFBP-3 and one or more GH stimulation tests) and MRI of the hypothalamic-pituitary region (8). If one parent is very short and GH deficient, a diagnosis of type 2 IGHD is almost certain, but it is still important to confirm this by genetic testing. In such cases, rhGH treatment in a substitution dose is highly effective in leading to rapid catch-up growth followed by a normal growth pattern and a normal adult height (6,9,14).

Infants with severe congenital GHD can present with neonatal hypoglycaemia, prolonged postpartum hyperbilirubinemia, elevated liver function tests and microphallus (1,4). Although data on blood glucose during the neonatal period were not available in our patient, the absence of reported neonatal seizures argue against a past history of hypoglycaemia. Neonatal hypoglycaemia is less frequent in isolated GHD than in MPHD (17,18).

While in this and similar cases the dominant inheritance and the classical phenotype made the diagnosis of type 2 IGHD straightforward, the diagnosis of less severe IGHD is much more challenging. In such cases the clinician has to make an assessment of the likelihood of IGHD based on the growth pattern, bone age delay, observations at physical examination and the results of the screening test (serum IGF-1) (6,8,9,19,20). If the likelihood appears sufficiently high, the next step is a GH stimulation test, which should be repeated if a low GH peak is observed, to exclude the possibility of false positive results (1,21). With regard to the growth pattern of children with GHD, height velocity can be very low in severe cases, particularly in the first years of life, but in other cases height SDS can stabilize for a number of years at or below the -2 SDS line of the population (but considerably below target height SDS), so that height velocity appears normal for the height SDS position. While in most cases height SDS is lower than TH SDS, the dominant form of IGHD, such as was present in our patient and other type 2 IGHD patients, can present with a height SDS close to the height SDS of one of the parents, so that for this subtype of IGHD the distance to TH is not a strong predictor (6,9,21).

Due to its pulsatile nature, physiological and pharmacological GH provocation tests are the key to assess GH secretion (9). The average GH response to various stimuli is slightly different and the level of adiposity is an important determinant of the GH peak, but usually a single cut-off is still used (1,21). Over time, this response moved upwards from 7 to 10 ng/mL (1,16), but due to the increased potency of GH standards a more rational cut-off may be at 7 ng/mL (22). Although few comparative studies have been performed, clonidine (through its stimulation of GHRH release) is thought to be a powerful stimulant for GH secretion, to a similar degree as insulin (1,23).

Each patient with a congenital GHD needs also to be evaluated with a brain MRI to search for anatomic abnormalities of the pituitary gland (24). MRI is an important tool to forecast future endocrine dysfunction, since individuals with abnormal pituitary anatomy are more likely to have or develop multiple endocrinopathies (25). MRI imaging in our patient demonstrated anterior pituitary hypoplasia, in line with the majority of patients with type 2 IGHD. The specific genetic diagnosis (splicing defect of *GH1*) increases the likelihood that with time other pituitary defects may develop (26).

It has been reported that 3-30% of individuals with isolated GHD have a genetic basis, but the likelihood of a genetic cause is considerably higher in children with a positive family history and/or in those with severe short stature (5). Mutations of relevant candidate genes have been identified in 11% of patients with severe IGHD and in frequencies as high as 38% in familial cases (13). Thus, genetic testing is recommeded in children with severe and/or familial IGHD (13,27,28). Children with proportionate short stature and a low peak GH after stimulation, without additional pituitary deficiency, should be considered for mutation screening for *GHRHR* and *GH1*. Another potential genetic cause is a *GHSR* mutation, although the wide phenotypic spectrum of published patients with such mutations do not allow for strong statements about their pathogenicity (28). While it was previously thought that GHD is almost always associated with a normal birth weight and length (1,19,21), it has recently become clear that average birth size of GHD infants is decreased (18). A positive family history of severe short stature in one of the parents strongly suggests an autosomal dominant inheritance pattern, which makes type 2 IGHD very likely, so that full gene sequencing of *GH1* is indicated, as was done in our patient (10,13,27,28,29).

In IGHD type 2, GH secretion is very low but usually still detectable and associated with heterozygous splice site, missense, splice enhancer mutations or intronic deletions in *GH1* (5,10,27,28,29). Most patients, such as ours, with type 2 IGHD have mutations within the first six nucleotides of intron 3 of *GH1*, resulting in skipping of exon 3. The result is the production of the 17.5-kDa isoform, which lacks amino acids 32-71 and, hence, the loop that connects helix 1 and helix 2 in the tertiary structure of GH. This isoform exerts a dominant negative effect upon secretion of the full-length GH molecule and may disturb the secretion of other pituitary hormones, such as TSH, LH and prolactin (5,10,29,30,31,32). Pre-treatment thyroid hormones, as well as other anterior pituitary hormones were normal in our patient. These values were also normal 18 months after start of rhGH treatment. The probability of having other pituitary hormone deficiencies in IGHD increases around puberty, and the first hormone to be affected is ACTH at around eight years of age (33). The normal results of pituitary testing in the patient’s father suggest that the risk of additional pituitary insufficiencies in this family may be limited.

In summary, we report a novel mutation in *GH1* leading to type 2 IGHD in an Indonesian child with a classical phenotype. Genetic testing is indicated in severe and or familial IGHD, particularly if one parent is also affected.

## Figures and Tables

**Table 1:**
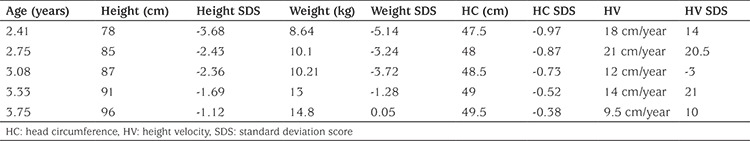
Summary of antropometric data of the patient during recombinant human growth hormone therapy
